# Auditory and cognitive contributions to recognition of degraded speech in noise: Individual differences among older adults

**DOI:** 10.1371/journal.pone.0331487

**Published:** 2025-09-04

**Authors:** Daniel Fogerty, Judy R. Dubno

**Affiliations:** 1 Department of Speech and Hearing Science, University of Illinois Urbana-Champaign, Champaign, Illinois, United States of America; 2 Department of Otolaryngology-Head and Neck Surgery, Medical University of South Carolina, Charleston, South Carolina, United States of America; Indiana University, UNITED STATES OF AMERICA

## Abstract

This study examined individual differences in how older adults with normal hearing (ONH) or hearing impairment (OHI) allocate auditory and cognitive resources during speech recognition in noise at equal recognition. Associations between predictor variables and speech recognition were assessed across three datasets that each included 15–16 conditions involving temporally filtered speech. These datasets involved (1) degraded spectral cues, (2) competing speech-modulated noise, and (3) combined degraded spectral cues in speech-modulated noise. To minimize effects of audibility differences, speech was spectrally shaped according to each listener’s hearing thresholds. The extended Short-Time Objective Intelligibility metric was used to derive psychometric functions that relate the acoustic degradation to speech recognition. From these functions, speech recognition thresholds (SRTs) were determined at 20%, 50%, and 80% recognition. A multiple regression dominance analysis, conducted separately for ONH and OHI groups, determined the relative importance of auditory and cognitive predictor variables to speech recognition. ONH participants had a stronger association of vocabulary knowledge with speech recognition, whereas OHI participants had a stronger association of speech glimpsing abilities with speech recognition. Combined with measures of working memory and hearing thresholds, these predictors accounted for 73% to 89% of the total variance for ONH and OHI, respectively, and generalized to other diverse measures of speech recognition.

## Introduction

The progressive loss of hearing sensitivity with age is so well established that it is codified by an international standard [1]. Hearing loss is also strongly associated with significant declines in speech recognition. Multiple factors are associated with declines in speech recognition with aging, involving peripheral, central auditory, and cognitive (i.e., cortical) factors [2]. Numerous studies over the last several decades have sought to detail the factors underlying individual differences in speech recognition declines (e.g., [3–14]). Several of these studies involved large test batteries of auditory and cognitive assessments designed to capture individual sources of variance in speech recognition. The results of these detailed analyses have broadly confirmed the hypotheses from the CHABA report [2] of the multifactorial nature of speech recognition declines, highlighting both auditory and cognitive factors, particularly in noise (e.g., [5]).

This prior work has generally focused on different levels of the auditory system. That is, declines in speech recognition may result from limitations in information processing at peripheral, central auditory, and/or cortical levels involving auditory and/or cognitive functions. However, the relative importance of certain functions may vary based on the listening context, demographic factors, and the listener’s overall auditory-cognitive profile. For example, cognitive processing may become more important and associated with individual differences in speech recognition due to limitations in auditory processing. Indeed, neuroimaging studies show this pattern of results with increased activity in brain areas, such as those involving error-monitoring or attention (e.g., [15,16]), as speech recognition declines (see meta-analysis by [17]). Listening to speech in noise also results in the recruitment of higher cortical networks [18,19].

Evidence such as this has been used to suggest that hearing loss, which reduces access to auditory speech cues, may increase the listening effort required during speech recognition as additional cognitive processing resources must be diverted to recover the degraded cues. However, in a systematic review, Ohlenforst et al. [20] found only equivocal evidence to support increased listening effort with hearing loss. One alternative hypothesis was that poorer auditory representation of speech cues with hearing loss limits the further recruitment of cognitive processes during speech recognition because less information is available for subsequent processing. This is supported by investigations that have shown poorer memory for word lists under degraded auditory conditions (e.g., [21,22]), suggesting that poorer sensory representations limit later processes related to the encoding and storage of information in memory. These findings have also been argued to support a limited capacity model for information processing (e.g., [23]), such that degraded input requires more processing, which diverts resources away from later cognitive stages of information processing, including memory (e.g., [24]). Whether it is the limited sensory information or limited cognitive resources that reduces subsequent down-stream processing is unclear. An alternative hypothesis is that a loss of signal quality may simply shift which processing resources are recruited during listening. That is, the relative balance of peripheral, central, and cortical functions contributing to speech recognition may become re-weighted due to hearing loss. This would suggest differences in the recruitment of resources, such as observed in the neuroimaging results, with perhaps minimal or no differences in overall listening effort, as observed in the meta-analysis [20].

All of these possibilities, whether related to limited capacity, information limitations, or resource reweighting, suggest that there is an interaction between the auditory and cognitive components that support speech recognition. Indeed, Wingfield and colleagues [23] argued for an interactive view of auditory and cognitive functions supporting speech recognition.

This study takes this interactive view and posits that hearing loss influences the allocation of auditory and cognitive resources during speech recognition. Specifically, this study was designed to assess how older adults with normal hearing and hearing loss recruit these abilities to support speech recognition. This is different from investigations of auditory-cognitive links in aging that demonstrate declines in general abilities (e.g., [25]). Rather, here the focus is on the involvement of these abilities during speech recognition tasks.

Based on prior studies [7,11], we hypothesized that a combination of auditory and cognitive-linguistic variables would predict speech recognition outcomes. Prior work has suggested that variance in speech recognition due to elevated hearing thresholds can be controlled using individualized frequency-specific gain [7,26], which we implemented here. Therefore, we expected greater contributions from cognitive variables than auditory variables. These prior studies explored individual differences among a single group of older listeners with a range of hearing thresholds, so it is unclear how auditory and cognitive contributions might vary based on the presence of hearing loss especially because hearing loss is expected to impose greater processing resource limitations. This could suggest more limited cognitive contributions for listeners with hearing impairment, or that variance in cognitive capacity could exert significant influence in speech recognition. Testing these hypotheses calls for investigation of individual differences in speech recognition based on hearing status.

Thus, the purpose of this study was to assess individual differences in the recognition of degraded speech for two groups of older adults: those with normal hearing and those with hearing loss. Similar to previous studies (e.g., [7,11]), we took an individual differences approach to investigate the association of various auditory and cognitive abilities to explaining speech recognition. Decades of research have highlighted the importance of assessing a broad range of auditory and cognitive abilities for explaining speech recognition across listeners (e.g., [3,4,26–29]) and in characterizing within-group individual hearing profiles [30–31], spanning domains of hearing sensitivity, psychophysical suprathreshold auditory processing, and cognitive measures such as attention and working memory. A challenge from previous reports has been a lack of consistency between the selection of measures and the types of speech recognition tasks and conditions assessed, resulting in inconsistencies in results and conclusions among studies. Therefore, the primary focus of this report is to identify the consistency of individual differences across several speech recognition experiments and tasks and the generalizability of these findings to other speech recognition tasks. A secondary focus of this report was to assess how individual differences might change according to the accuracy of speech recognition, which also affects cognitive effort (e.g., [32]). Such effects might change the recruitment or allocation of auditory and cognitive processing resources [24]. Here we assessed the association of auditory and cognitive abilities at a range of speech recognition thresholds (SRTs). This allowed us to assess differences in resource recruitment across recognition levels, and to compare resource recruitment between older adults with normal and impaired hearing when assessed at equal recognition. As older adults with hearing loss typically have poorer speech recognition compared to those with normal hearing, this step was necessary to determine if group differences in resource allocation were related to hearing loss or recognition.

For the current study, all participants completed a series of baseline auditory and cognitive measures (predictor variables), followed by three experiments of recognition of degraded speech in noise (speech outcome variables). Regression models were used to assess the contribution of baseline abilities to explain speech recognition for each of the three experimental datasets and at three SRTs. Additional speech recognition measures were used to assess the generalizability of these findings.

## Methods

### Participants

A total of 40 older adults were included in this analysis: 20 older adults with normal hearing (ONH; 17F, 3M; mean 67 years, 60–74 years) and 20 older adults with hearing loss (OHI; 13F, 7M; mean 72 years, 60–85 years). Participants completed the Mini Mental Status Exam (MMSE) [33] with all participants scoring ≥ 26 (mean = 29 out of 30). All participants had corrected vision at or better than 20/40 on a Snellen chart, except for one participant in each group with scores of 20/50. An additional 64 younger adults with normal hearing (46 F, 18 M; mean 22 years, 18–38 years) also completed all tasks. Results on the individual speech recognition tasks from these younger listeners are detailed in previous reports [34–36] and are not discussed here given the focus on individual differences among older adults. However, so that variance in scores due to age and hearing loss are captured in the current report, factor analyses reported here were conducted with the full participant sample (N = 104) to increase power and better capture individual variance associated with age. The experimental protocol was approved by Institutional Review Boards of the University of South Carolina and the Medical University of South Carolina, with subsequent data use procedures approved by the University of Illinois. Written informed consent was obtained for all participants. Participants were reimbursed for their participation. Recruitment of the older participants reported here occurred between December 4^th^, 2017 and December 14^th^, 2018.

## Predictor variables

### Auditory measures

**Fig 1:**
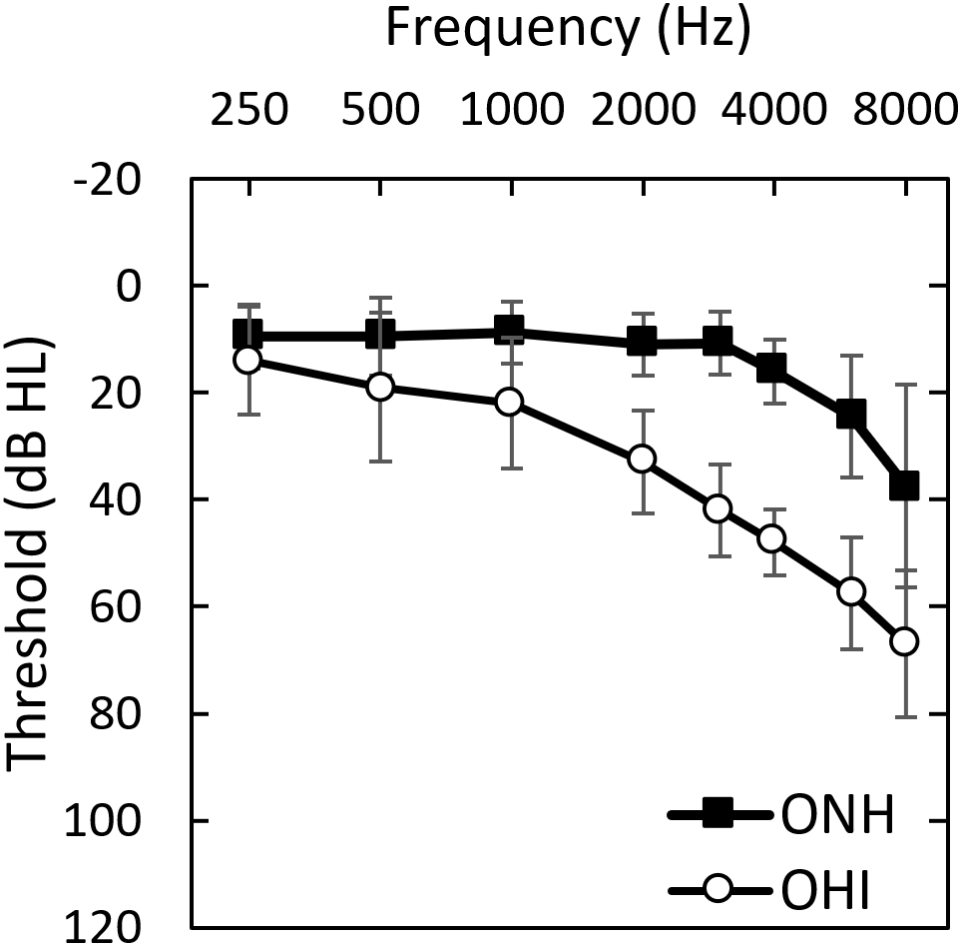
Mean audiograms for the two listener groups. Error bars display ± 1 standard deviation.

*Pure-tone hearing thresholds.* All participants completed pure-tone threshold testing at standard audiometric frequencies between 250–8000 Hz. Audiometric thresholds for the two listener groups are displayed in Fig 1.

*Psychoacoustic measures of modulation detection and modulation interference using speech-modulated noise.* Speech temporal-envelope modulations, adaptively compressed using an exponential function, were imposed on target noise bands (i.e., 500 Hz and 3150 Hz) of a speech-spectrum 500-ms noise. Psychoacoustic tasks presented stimuli in a three-interval two-alternative forced choice task. *Modulation detection* required participants to indicate the interval containing the target band modulation compared to the interval with two unmodulated noise bands. *Modulation interference* required participants to indicate the interval containing the target band modulation with concurrent uncompressed speech modulation in the non-target band, which was present in the target and comparison intervals. Additional details regarding the methods and data reduction results for this task can be found in S1 Appendix.

*Speech glimpsing* refers to the ability to make use of partial acoustic information to understand speech. Glimpses are time-frequency regions in which the speech occurs at relatively favorable signal-to-noise ratios (SNRs). Participants’ open-set sentence recognition abilities were measured in two maskers (steady-state or unmodulated speech-shaped noise, SSN) and speech-modulated noise (SMN). Speech glimpsing was operationally defined as a fluctuating masker benefit (FMB), calculated as the difference in keyword accuracy for speech presented in SMN and SSN (that is, FMB = SMN – SSN). SSN was created to match the long-term average speech spectrum for concatenated IEEE sentences [37] prior to shaping. The SMN was created by amplitude modulating the SSN by the wideband temporal envelope of the concatenated sentences, extracted using half-wave rectification. Amplitude modulations were low-pass filtered using a 4^th^ order Butterworth filter with a cutoff modulation frequency of 16 Hz. A random selection of noise with a 100-ms onset/offset padding was added to the speech on every trial. To reduce individual variability, SSN was always tested prior to SMN because speech recognition in SMN is an easier task due to speech glimpsing (e.g., [38,39]).

### Cognitive process-based measures

In order to index a broad range of cognitive processes, an in-person, computerized version of the Brief Test of Adult Cognition by Telephone (BTACT) [40] was created using speech recorded by a female speaker. This test was designed to be sensitive to cognitive changes in normal aging. Seven subtests were included: Short Recall, Delayed Recall, Category Fluency, Auditory Backward Digit Span, Backward Counting, and Number Series Completion.

*Short and Delayed Recall* subtests assessed immediate and delayed episodic memory. These subtests auditorily presented a list of 15 words, spoken at a rate of one word per second. Participants recalled as many words from the list as possible within 1 minute. Short Recall involved repeating the word list immediately after presentation. The Delayed Recall subtest prompted the participants to recall the same word list from the Short Recall task, but the prompt was provided after completing all other BTACT subtests.

*Category Fluency* is a test of verbal fluency and executive function that requires participants to name as many animals as possible within one minute. Scores indicate the number of unique responses within the allowed time limit.

*Auditory Backward Digit Span* assessed working memory, requiring listeners to recall a list of digits in backward order. Digits were presented at the rate of one digit per second in sequences ranging from two to eight. A set of two trials were presented at each sequence length. The test was discontinued when the participant missed both trials in a set and the score was the number of digits in the longest sequence correctly reproduced.

*Number Series Completion* assessed inductive reasoning. Participants controlled the rate of presentation for a series of five numbers. Following the presentation of the fifth number, participants were prompted to indicate a sixth number that would best complete the sequence. Scores indicated the number of sequences completed correctly out of five.

*Backward Counting* (30-SACT subtest) was a measure of processing speed that involved counting backward from 100 as quickly as possible. The score was the number of correctly repeated numbers within 30 seconds.

In addition to these auditory measures from the BTACT, three additional cognitive measures were included in the visual modality.

*Visual Backward Digit Span* replicated the Auditory Backward Digit Span procedures using a visual presentation of digits one at a time on the screen, each digit visible for 1 second.

*Connections Test* [41] is a version of the Trail Making Test in which participants draw lines to connect numbers and/or letters in sequence. Participants first completed drawing lines between ascending numbers and alphabetically sequenced letters in two different trials. They then completed two trials where they were required to alternate between ascending sequences of numbers and letters, beginning with either a number or a letter, respectively. The number of circles properly sequenced within 20 seconds was counted. The final score was the average of the two alternating sequence trials.

*Stroop Color-Word Mismatch* [42] assessed inhibitory control. Participants completed three training trials: (1) reading color words printed in black ink; (2) naming the color of ink for a “xxxx” text sequence, and (3) reading color words printed in colored ink that matched the target word. Following these three trials, participants were presented with a page with color words printed in color ink that mismatched the written words. Participants were required to name the color of the ink while ignoring the written words. The score was the number of correctly identified colors within 45 seconds.

### Cognitive-linguistic processing

In addition to the process-based measures of cognitive function, two additional measures were associated with product-based measures of cognitive-linguistic processing: vocabulary knowledge and linguistic closure.

*Vocabulary Knowledge* was assessed using a computerized version of the Peabody Picture Vocabulary Test Fourth Edition (PPVT-IV) [43]. Participants heard words recorded by a female speaker and selected a corresponding picture using a touch screen interface. Standard test procedures were used, and raw scores were saved for analysis.

*Linguistic Closure* was assessed using text recognition threshold testing (e.g., [44]. In the current implementation, the text of IEEE sentences was presented with periodic portions of text deleted. Four proportions of text preservation were tested at 60, 70, 80, and 100%. Logistic functions were fit to the data to derive the text proportion threshold for 50% correct keyword recognition (SRT50), which was the outcome variable of interest for the current investigation. Results from this test were previously reported [45]. All participants scored ≥ 94% on the 100% condition, indicating sufficient visual acuity for the visual tasks.

### Speech recognition outcome variables

Speech recognition scores from three previously reported studies were used as the outcome variables of interest. To examine reproducibility, these studies were examined independently to determine if the same pattern of predictors would be observed across studies. All three studies examined the recognition of speech filtered in the temporal modulation domain to examine the contribution of different temporal speech modulation rates to the recognition of degraded speech in noise. As a final measure of generalizability, more standard measures of speech recognition without filtering in the temporal modulation domain were examined. While this analysis provides an indication of whether the same predictors consistently emerge across studies, it is important to note that these studies are not completely independent observations as the same listeners participated in all studies. Therefore, the degree of replication across datasets should be considered as a measure of the reliability of individual differences observed in our sample population.

### Experimental datasets for the recognition of temporal-envelope filtered speech

*Dataset #1: Spectrally reduced speech (D1*_*SR*_). Fifteen acoustic conditions of spectrally reduced speech were analyzed from [35]. TIMIT sentences [46] were bandpass filtered into 18 one-third octave bands. The Hilbert envelopes were extracted from each band. Envelopes were bandpass filtered into two modulation bands: 0–8 Hz and 8–16 Hz. Filtered envelopes were combined with the original spectral components and summed across bands to re-synthesize the original speech sample with reduced temporal modulation cues. Contribution of the modulation depth was assessed by scaling the consonant or vowel levels by a factor of 0.5, 1.0, or 2.0. This resulted in amplitude compression/expansion of the consonant–vowel intensity ratio using the TIMIT phonetic markings. Two modulation bands, two manipulated segments (consonants/vowels), and three segment level settings (0.5, 1.0, 2.0), plus three control conditions of the full sentence limited with temporal modulations filtered at 0–8, 8–16, or 0–16 Hz were tested. This resulted in 15 acoustic conditions tested in the presence of a 2 dB SNR signal-correlated noise, which primarily reduced the spectral content of the speech.

*Dataset #2: Noise-masked speech (D2*_*NM*_). Sixteen acoustic conditions of temporal-envelope filtered speech (IEEE sentences) and noise were analyzed from Experiment 1 of [36]. As in D1_SR_, sentences were passed through 18 one-third octave bands. Hilbert envelopes were then extracted, temporally filtered into a specified speech modulation band, recombined with the original spectral components, and summed across bands. This processed speech was then presented in a steady-state noise (SSN) that matched the long-term average spectrum of the target speech, or a speech-modulated noise (SMN) that modulated the SSN by the temporally filtered Hilbert envelope of a different sentence spoken by the target talker. The temporal envelope for SMN modulation was exponentially modified, E(t)^K^, (K = 0.5, 1.0, or 2.0; with 1.0 equal to the original speech modulation) to compress or expand the modulation depth (as in [34,47,48]. Four baseline conditions tested included 0–16 Hz temporally filtered speech in unmodulated SSN and in 0–16 Hz SMN at K = 0.5, 1.0, and 2.0. The remaining 12 conditions tested two speech modulation bands (0–8 Hz, 8–16 Hz), in SMN with two noise modulation bands (0–8 Hz, 8–16 Hz), at three noise modulation depths (K = 0.5, 1.0, and 2.0). This resulted in a total of 16 conditions of temporally reduced speech in modulated noise.

*Dataset #3: Spectrally reduced and noise masked speech (D3*_*SR+NM*_). A final set of 16 acoustic conditions was selected from Experiment 2 of [36]. This experiment was identical to the speech and noise conditions just described in D2_NM_ (i.e., speech modulation (0–8 Hz, 8–16 Hz) × SMN modulation (0–8 Hz, 8–16 Hz) × noise modulation depths (K = 0.5, 1.0, and 2.0) + 0–16 Hz speech in SSN, or 0–16 Hz SMN at K = 0.5, 1.0, 2.0), with one exception. During speech processing, instead of recombining filtered Hilbert envelopes with the original spectral components of the speech, it was combined with the spectral components of the SSN. This resulted in an 18-channel noise-vocoded speech signal that was used as the target.

### Generalized measures of speech recognition

Three additional speech recognition tasks were also measured to determine the generalizability across measures of speech without temporal-envelope modulation filtering.

*Interrupted speech.* Speech recognition thresholds for interrupted IEEE sentences were selected from [45]. Speech was interrupted at 2 Hz, which represents the rate most affected by advancing age [49,50]. Speech was tested at fixed proportions of speech preservation (i.e., duty cycles) and fit with a logistic function to determine the duty cycle threshold for 50% and 80% correct recognition.

*Competing talker.* Following procedures from [51], the Coordinate Response Measure (CRM) [52] sentences were presented in the presence of a single competing talker at 0 dB SNR under selective and divided attention conditions. CRM sentences are in the form: “Ready {call sign} go to {color} {number} now.” Three male and three female speakers were selected. One of the six talkers was randomly selected on each trial as the target talker. The masker talker was selected from the three talkers of the different sex. The call sign was used to cue the target talker and was presented as text onscreen prior to the start of the trial for selective attention or after the presentation of the trial for divided attention. Participants responded using a touchscreen interface to indicate the number and color of the sentence. Selective and divided attention conditions were tested in separate blocks of 32 trials each.

*Speech in babble.* Fifty sentences from the Revised Speech Perception in Noise (R-SPIN) [53,54] test were tested in 12-talker babble at 0 dB SNR. Participants responded by repeating the final word in the sentence. Half of the sentences had low contextual predictability and half of the sentences had high predictability for the final word. Two scores were recorded, corresponding to the two sentence types.

### General procedures

All participants completed the battery of tests described above (summarized in Table 1) over at least five test sessions. Participants completed all testing in a sound-attenuating booth and listened to stimuli at a sampling rate of 48,828 Hz via one of a pair of Sennheiser HDA 200 headphones following a TDT System III digital-to-analog processor (RP2/RX6) and headphone buffer (HB7/HB5). Presentation was monaural to the right ear, unless target sensation levels (SLs) were better obtained using the left ear (3 ONH, 14 OHI). To ensure audibility of the speech materials (i.e., > 15 dB SL) through at least 4000 Hz, including all speech presented during cognitive testing (e.g., task instructions, number or word stimuli, etc.), all listeners received frequency-specific gain based on individual hearing thresholds (i.e., spectral shaping). To limit the contribution of reduced audibility in the higher frequencies, all stimuli were subsequently passed through a low-pass, linear phase, finite-impulse-response, 128th-order filter with a cutoff of 5,623 Hz. All auditory stimuli were calibrated to be presented at 70 dB SPL, with a mean presentation level of 82 dB SPL for OHI listeners following spectral shaping. Thus, all test materials – auditory and cognitive – were presented at SLs that ensured audibility for each participant.

**Table 1:** Summary table of experimental measures, including domains, tasks, and variables.

Domains	Tasks	Variables
*Predictor Measures*		
Auditory measures	Pure-tone hearing thresholds Speech modulation detection Speech modulation interference Speech glimpsing	8 test frequencies (dB HL) 2 modulation bands (k threshold) 2 modulation bands (k threshold) Fluctuating masker benefit
Cognitive process-based measures	Short recall Delayed recall Category fluency (semantic) Number series completion Backward counting Backward digit span (auditory) Backward digit span (visual) Connections Stroop	Number of words recalled Number of words recalled Number of words Accuracy Number reached Span length Span length Number sequenced Number identified
Cognitive-linguistic measures	Vocabulary knowledge Linguistic closure (visual)	PPVT raw score Duty cycle at 50%
*Outcome Measures*		
Recognition of temporal-envelope filtered speech	Spectrally reduced speech (D1_SR_) Noise-masked speech (D2_NM_) Spectrally reduced and noise-masked speech (D3_SR+NM_)	15 degraded speech conditions 16 degraded speech conditions 16 degraded speech conditions
Recognition of generalized speech measures	Interrupted speech Competing talker (attention) Speech-in-babble	Duty cycle at 50% and 80% Accuracy (selective/divided) Low/high context predictability

PPVT = Peabody Picture Vocabulary Test; SR = spectrally reduced; NM = noise masked.

All measures of speech recognition involved participants repeating sentences, unless otherwise stated (e.g., CRM closed-set responses). These open-set responses were live-scored and recorded. Participants were encouraged to guess and no feedback was provided. A response was scored as correct if the participant repeated each keyword exactly (e.g., without missing or extra phonemes). Finally, while some measures included stimuli from the same speech corpus, no sentence was ever repeated across the complete set of predictor and outcome measures.

## Results

### Dimension reduction of predictor variables

Significant redundancy was expected among the various sets of variables (see Table 1 for a summary of auditory, cognitive process, and cognitive-linguistic variables). To examine the contribution of the underlying constructs corresponding to these auditory and cognitive variables, it was necessary to first reduce the dimensionality of the data.

#### Hearing thresholds.

Regarding hearing sensitivity, thresholds at eight pure-tone frequencies (250, 500, 1000, 2000, 3000, 4000, 6000, and 8000 Hz) were measured in the test ear. Because of the large number of threshold measures (eight) and their lack of independence, principal components analysis (PCA) was used to reduce the dimensionality of the data while retaining much of the original variance. Hearing thresholds at eight frequencies were entered into the analysis to extract factors with eigenvalues greater than 1. A single factor emerged that explained 79.5% of the variance across frequency. All communalities were greater than 0.50 (7 out of 8 were > 0.70, KMO = .89). This analysis resulted in a single “Audiogram PCA” score strongly correlated with the eight-frequency pure-tone average (250–8000 Hz; ONH: r = .98, OHI: r = .98) reflective of hearing loss severity.

#### Cognitive process-based measures.

A similar dimension reduction step was employed on the nine cognitive process-based measures. Raw scores were first transformed to z-scores and then subjected to PCA to extract factors with eigenvalues greater than 1. A three-factor solution emerged that explained 77% of the variance. All communalities were greater than 0.58 (7 out of 9 were > 0.78, KMO = .79). The pattern matrix following varimax rotation with kaiser normalization is provided in Table 2. Principal components were interpreted as indexing episodic memory, working memory, and interference.

**Table 2:** Component weights for each cognitive process-based measure for the rotated PCA factor solution.

Measure	Episodic Memory	Working memory	Interference
Short Recall	**.911**	.210	.085
Delayed Recall	**.892**	.154	.169
Category Fluency	**.647**	**.420**	.214
Number Series	.335	**.661**	.361
Auditory Backward Digits	.240	**.841**	.154
Visual Backward Digits	.100	**.880**	.086
Backward Counting	.352	**.588**	.332
Connections	.159	.251	**.870**
Stroop	.142	.147	**.911**

Weights > .4 are shown in bold. PCA = principal components analysis.

#### Individual differences among older adults on speech recognition measures.

Individual differences were assessed through three primary methods of analysis. The first analysis examined the contribution of predictor variables to different levels of performance on the psychometric speech recognition function. The second analysis examined these contributions on a macro level to summarize the main contributions. The third analysis determined the extent to which these patterns generalized to other measures of speech recognition.

#### Contribution across performance levels.

From the initial reports of these speech recognition measures [34–36], clear group differences between ONH and OHI were observed with the OHI group typically performing poorer. Prior work has clearly detailed that cognitive recruitment may vary with the difficulty of the speech recognition task (see [24] for discussion). In this analysis the goal was to characterize how the two groups of older adults recruited auditory and cognitive abilities at equal levels of task difficulty for speech recognition. Furthermore, we sought to identify if these patterns changed as the task difficulty decreased, potentially by freeing additional cognitive resources or by increasing signal fidelity to enable higher levels of speech and language processing.

#### Preparatory analyses.

**Fig 2:**
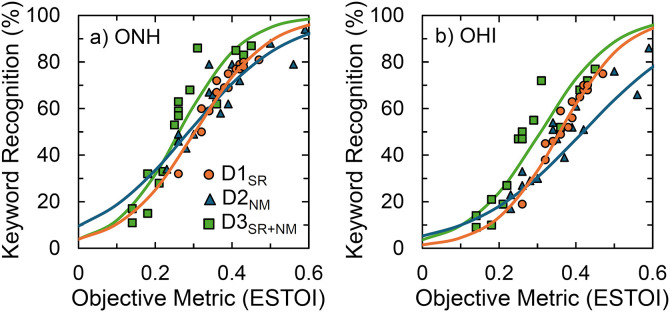
Speech recognition psychometric functions. Lines indicate each of the three datasets for (a) ONH and (b) OHI groups.

The first step in this analysis was to derive the psychometric functions for each of the three speech recognition tasks. The psychometric functions were previously calculated for the two noise masked datasets (D2_NM_ and D3_SR+NM_) in [36]. Using this method, psychometric functions were also calculated for D1_SR_. As all datasets used different and multiple forms of distortion, it was important to base psychometric functions on a single objective measure of speech distortion. For this reason, all stimuli were analyzed using the Extended Short-Time Objective Intelligibility metric (eSTOI) [55]. The eSTOI compares the spectro-temporal modulation envelopes of the clean and degraded speech signals over short-time segments to produce a similarity measure, with values less than 1.0 indicating the degree of acoustic distortion. The 15–16 experimental conditions were defined according to eSTOI units and logistic functions were fit to the individual data (see Fig 2), which explained 82% − 93% of the total variance on each dataset for the two groups. Speech recognition thresholds (SRT) were calculated at 20%, 50%, and 80% correct (i.e., SRT_20_, SRT_50_, SRT_80_, respectively). Overall, Fig 2 demonstrates that OHI listeners required greater speech preservation (i.e., higher eSTOI values) than ONH listeners to obtain comparable speech recognition, as indicated by the rightward shift of the psychometric functions in panel (b) for the three datasets. Full analysis details can be obtained from the prior dataset reports [35,36].

Next, an initial exploration of the correlational matrix for each group was examined for the seven potential predictor variables (Table 3). This included the principal components derived during factor analysis along with an additional auditory measure and two cognitive-linguistic measures for a total of eight predictors (i.e., Audiogram PCA, psychoacoustic speech modulation PCA, three cognitive PCAs, speech glimpsing, vocabulary knowledge, and linguistic closure). Only four measures consistently demonstrated significant correlations with any of the outcome variables for either group. These involved two auditory measures: Audiogram PCA and glimpsing; and two cognitive measures: working memory PCA and vocabulary knowledge. Each of these measures had significant correlations on 13–16 comparisons, with average absolute r-values ranging from 0.43 to 0.82 (p < .05). These four predictor variables were selected for the full analysis. Two other measures, linguistic closure and interference, were significant for one or two comparisons, respectively, with absolute r-values from 0.45 to 0.51 (p < .05). These variables were excluded due to their relatively low associations across the range of measures, particularly considering the multiple comparisons.

**Table 3:** Correlation matrix of predictor variables with the three speech recognition outcome datasets.

		D1_SR_			D2_NM_			D3_SR+NM_		
Predictor	Group	SRT_20_	SRT_50_	SRT_80_	SRT_20_	SRT_50_	SRT_80_	SRT_20_	SRT_50_	SRT_80_
Audiogram PCA	ONH	**0.43**	**0.52**	**0.55**	**0.52**	**0.60**	**0.49**	**0.64**	**0.56**	0.42
	OHI	**0.64**	**0.56**	0.41	**0.74**	**0.82**	**0.82**	**0.72**	**0.76**	**0.68**
Speech	ONH	0.14	0.25	0.24	0.17	0.30	0.32	0.32	0.33	0.29
Modulation PCA	OHI	−0.20	−0.29	−0.33	−0.24	−0.26	−0.26	−0.29	−0.26	−0.20
Glimpsing	ONH	−0.30	**−0.46**	**−0.45**	−0.18	−0.39	**−0.47**	**−0.46**	**−0.50**	**−0.47**
	OHI	**−0.74**	**−0.74**	**−0.66**	**−0.67**	**−0.70**	**−0.68**	**−0.64**	**−0.67**	**−0.61**
Working	ONH	−0.37	**−0.49**	**−0.50**	−0.33	−0.44	−0.43	−0.43	**−0.57**	**−0.61**
Memory PCA	OHI	**−0.67**	**−0.58**	**−0.48**	**−0.61**	**−0.65**	**−0.63**	**−0.58**	**−0.70**	**−0.71**
Episodic	ONH	0.03	−0.18	−0.31	−0.06	−0.25	−0.33	−0.15	−0.20	−0.22
Memory PCA	OHI	0.21	0.12	0.05	0.14	0.14	0.12	0.12	0.09	0.04
Interference PCA	ONH	0.14	0.17	0.22	0.25	0.12	−0.04	−0.08	0.03	0.12
	OHI	−0.32	−0.30	−0.29	−0.42	**−0.48**	**−0.51**	−0.29	−0.40	−0.44
Vocabulary	ONH	**−0.46**	**−0.64**	**−0.66**	−0.44	**−0.61**	**−0.59**	**−0.56**	**−0.64**	**−0.62**
Knowledge	OHI	**−0.60**	**−0.55**	**−0.49**	−0.38	**−0.48**	**−0.54**	**−0.45**	**−0.61**	**−0.67**
Linguistic	ONH	**0.45**	0.42	0.36	0.20	0.34	0.37	0.30	0.25	0.17
Closure	OHI	−0.12	−0.16	−0.15	−0.12	−0.12	−0.13	−0.12	−0.08	−0.04

Bold = significant at p < .05. PCA = principal components analysis; SR = spectrally reduced; NM = noise masked; ONH = older normal hearing; OHI = older hearing impaired.

**Fig 3:**
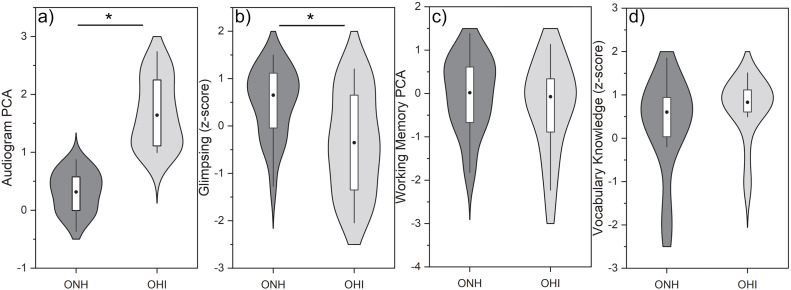
Violin plots for the four predictor variables. Center box plots display interquartile range and median (dot). * p < .01.

The distributions for these four selected predictor variables are shown in Fig 3 for the two listener groups. Independent samples t-tests confirmed that OHI, compared to ONH, had poorer thresholds (t(39) = −8.4, p < .001, d = −2.6) and reduced speech glimpsing (t(39) = 3.3, p = .002, d = 1.0). Comparisons for the two cognitive measures (right two panels) indicated no significant group differences for either working memory (t(39) = 1.2, p = .23) or vocabulary knowledge (t(39) = −1.4, p = .16). This indicates that while the groups were defined by differences on the auditory predictor variables, they were matched on the cognitive predictor variables.

##### Explanatory analysis.

Next, a dominance analysis [56,57] was conducted to determine the relative contribution of these four predictor variables to speech recognition. This follows a similar procedure previously conducted by Humes et al. [7] to model global speech recognition in older adults. Dominance analysis measures the importance of each predictor variable as defined by the amount of variance accounted for that variable alone and in all possible combinations of the other predictor variables from the linear regression model. This dominance analysis was conducted for each of the nine outcome variables (i.e., three SRTs for three datasets). Results of the full dominance analysis (i.e., all comparisons) are provided in S1 Supporting Information.

**Fig 4:**
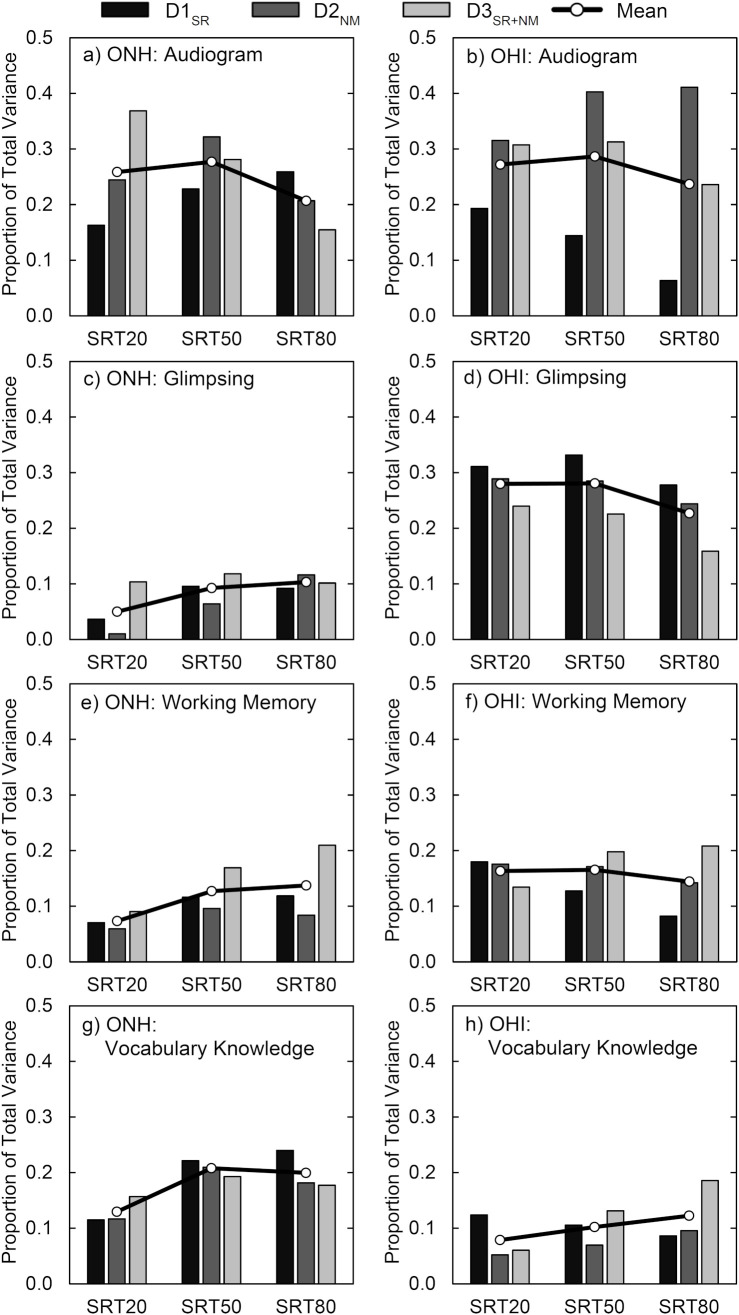
Results of the dominance analysis. Plotted is the proportion of total variance explained for the three datasets at each of the three SRTs for ONH and OHI groups. Rows display results for each of the four predictor variables and columns display results for the two groups. Bold lines connect the average dominance of three datasets across SRTs.

Fig 4 plots the general dominance (i.e., total variance in speech recognition) obtained for each dataset (i.e., bars) and listener group (i.e., columns) for each of the four predictors from the dominance analysis (i.e., rows). Each row in the figure is associated with one of the four predictors, with the left panels displaying results for ONH and the right panels for OHI. Bars within each plot display the general dominance for that predictor variable for each dataset at each SRT. The mean dominance, averaged across the datasets, is plotted as the solid line.

**Fig 5:**
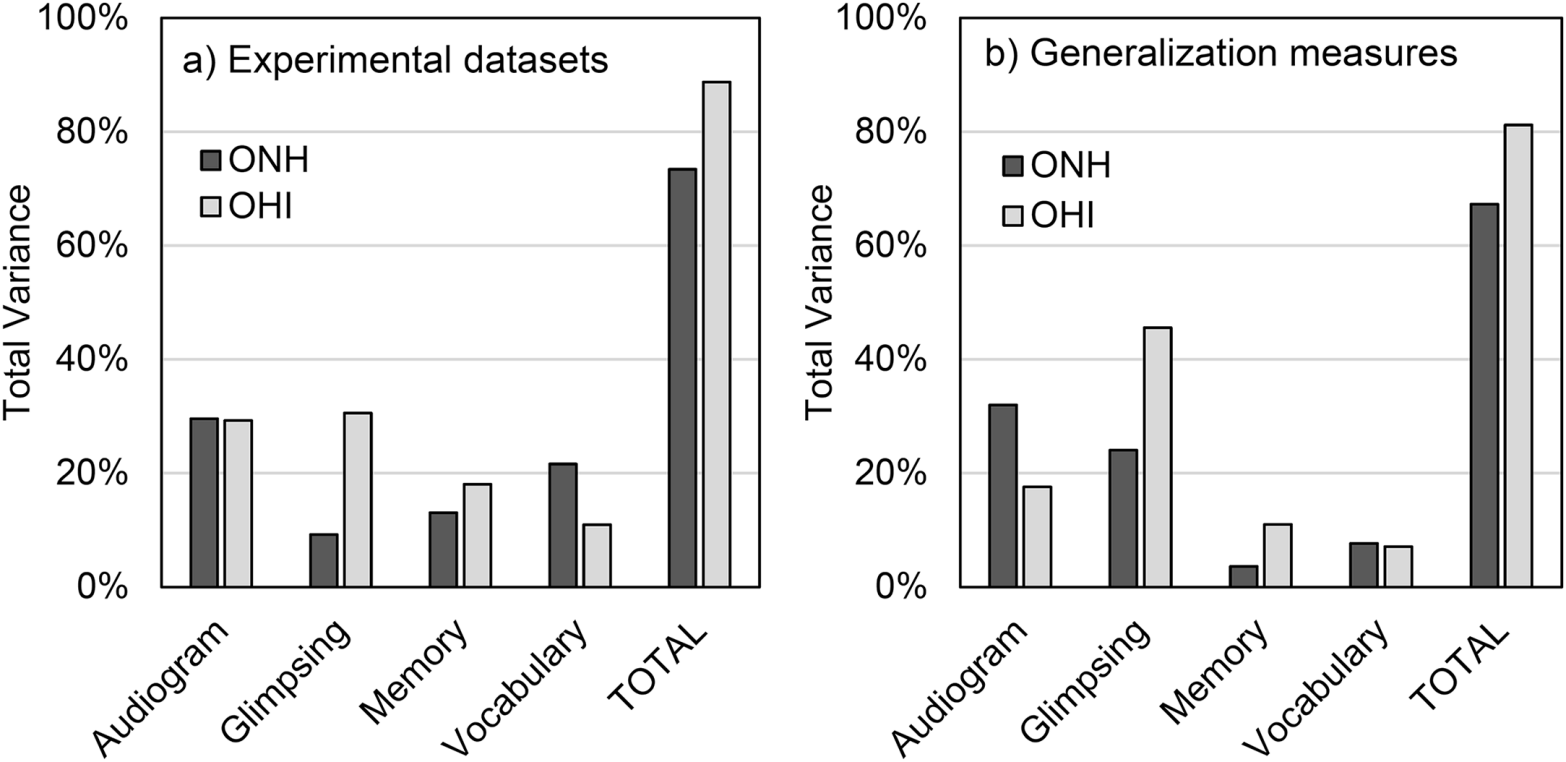
Overall and generalized dominance. Percent of total variance explained for ONH and OHI groups on the principal components derived from (a) the three experimental speech datasets, and (b) the generalization speech measures.

Some general variance is observed across datasets and at the different SRTs. However, the most predominant differences observed are between groups and across the different predictor variables (i.e., individual panels in Fig 5). Notable are the group differences for glimpsing, which provided minimal contribution for ONH compared to OHI speech recognition. An opposite pattern was observed for vocabulary knowledge, with greater contributions for ONH across datasets. Azen and Budescu [56] describe a hierarchy of dominance from complete dominance (dominant for all comparisons) to conditional dominance (dominant for all model sizes, i.e., number of predictors entered) to general dominance (dominant based on the overall average, as plotted in Fig 4). Most notable is that for OHI listeners, complete dominance (i.e., dominance for all comparisons) was observed for glimpsing in D1_SR_ involving spectral distortion across all SRTs, which switched to complete dominance of the Audiogram PCA in D2_NM_ and D3_SR+NM_ involving noise masking (except for one comparison at SRT_50_ in D2_NM_ in which the Audiogram PCA demonstrated conditional dependence). No other comparisons demonstrated complete dominance across SRTs.

#### Contribution to overall performance.

In the previous analyses, commonalties were observed across datasets and SRTs, with primary differences observed between groups for the different predictor variables. Therefore, this next analysis was conducted to summarize these general patterns across all datasets. Toward this aim, factor analysis was conducted across the three SRTs from the three datasets (9 values). Again, as this analysis was conducted at equal SRTs, the overall accuracy of speech recognition was matched between the two groups. A single factor was obtained that explained 82% of the variance across all three datasets at the specified SRTs. Communalities were all greater than 0.64 (KMO = .73).

A dominance analysis (Fig 5a) was again conducted to explain the variance on this single principal component reflecting overall speech recognition using the four predictor variables. This model explained 73% of the variance for ONH and 89% of the variance for OHI. For ONH, the Audiogram PCA (30%) followed by vocabulary knowledge (22%) were the dominant predictor variables with minimal weight given to glimpsing (9%). For OHI, Audiogram PCA (29%) and glimpsing (31%) had high general dominance, with vocabulary knowledge at 11%. Working memory PCA had similar dominance for the two groups (ONH:13%; OHI: 18%). While the combination of these predictor variables explained a large amount of the variance on this general speech recognition component, linguistic processing factored heavily into ONH performance and access to speech information through glimpsing was a dominant factor for OHI (with minimal contribution for ONH).

#### Generalization to diverse measures of speech recognition.

Of interest is whether this pattern of results generalizes to other speech materials that did not involve the significant signal processing employed for temporal-envelope filtered speech in the primary datasets. Toward this end, analysis using the generalized speech recognition measures (see II.C.2) was conducted. These generalized measures presented speech in the presence of multitalker babble (high and low sentence predictability), a competing talker (selective and divided attention), or under conditions of speech interruption (SRT_50_ and SRT_80_). These were highly diverse measures that used different speech corpora consisting of open-set and closed-set responses, varying in linguistic and cognitive predictability, manipulating attentional demands, and employing different forms of speech masking. A total of six conditions were z-transformed and entered into a factor analysis. This resulted in a single principal component representing generalized speech recognition abilities under adverse conditions, accounting for 59% of the variance. Five of six communalities were greater than 0.51, with one at 0.36 (KMO = .82).

Dominance analysis (Fig 5b) was conducted again on this single principal component. Total variance accounted for by all four factors was 67% for ONH and 81% for OHI listeners. For ONH listeners, the Audiogram PC was completely dominant for all comparisons, accounting for 32% of the total variance, followed by glimpsing at 24%. For OHI, speech glimpsing was completely dominant for all comparisons, accounting for 46% of the variance, followed by the Audiogram PCA at 18%. The importance of speech glimpsing abilities is consistent with the use of interrupted speech (one of the three primary generalization measures) as a model stimulus for studying glimpsing [58]. The minimal weight on vocabulary knowledge and working memory for both ONH and OHI may also be expected given the closed-set keyword responses from the CRM and final word recognition for R-SPIN. Overall, these results are in large agreement with the overall dominance analysis conducted over the three primary datasets (Fig 5a). The pattern of results confirms the high importance of glimpsing for the OHI group, while it may provide some contribution to ONH speech recognition when provided minimal linguistic constrains (e.g., closed-set testing). This is in addition to contributions of the Audiogram PCA for both groups.

## Discussion

This study reports individual differences among older adults in recruiting auditory and cognitive abilities while listening at equal levels of task difficulty for speech recognition in noise. Patterns of individual differences were compared between two groups of older adults, one with normal hearing and the other with hearing loss. The consistency of these individual difference patterns was investigated across multiple datasets and for generalized measures of speech recognition under diverse listening conditions.

Using robust multiple regression dominance analysis methods, the results demonstrate systematic differences for ONH and OHI listeners in the contributions of auditory and cognitive variables to speech recognition in various adverse listening conditions. Speech recognition by ONH listeners was characterized by recruitment of vocabulary knowledge skills. In contrast, OHI listeners have reduced speech glimpsing abilities, which interferes with recruiting cognitive-linguistic processes to facilitate speech recognition. This was observed even though (1) OHI received frequency-specific gain fit to their individual hearing thresholds, (2) performance between OHI and ONH groups was matched at equal SRTs, and (3) OHI and ONH groups were matched according to working memory PCA and vocabulary knowledge abilities.

**Fig 6:**
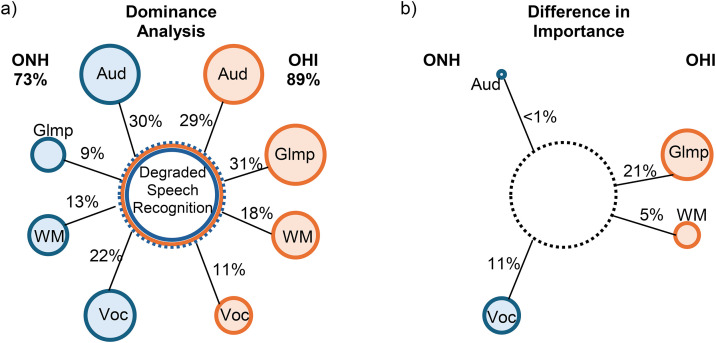
Schematic of the dominance analysis. (a) The total variance explained in degraded speech recognition for ONH and OHI listener groups and proportions of the total variance associated with each predictor. (b) The difference in importance between groups in variance explained by each predictor. Aud = Audiogram PCA, Glmp = speech glimpsing, WM = working memory PCA, Voc = vocabulary knowledge.

Fig 6a displays a schematic summarizing the results of the dominance analysis that was conducted across the three datasets at three SNRs. The bubble chart indicates the variance explained by each predictor along with the total variance explained in degraded speech recognition for each group. Larger bubbles indicate greater variance explained, with the numerical amount labeled on the connector lines. To facilitate between-group comparisons, the difference in variance explained between ONH and OHI groups for each predictor is displayed in Fig 6b. This figure can be taken as a simplified conceptualization of the primary study results. Larger bubbles on the left side indicate greater importance for ONH listeners, while larger bubbles on the right side indicate greater importance for OHI listeners. This analysis suggests that ONH and OHI place similar importance on working memory and on auditory abilities indexed by the audiogram. In contrast, large differences are observed in vocabulary knowledge for ONH and glimpsing for OHI.

These findings suggest that at equal levels of speech recognition task difficulty and equal SRTs, ONH and OHI listeners recruit different auditory and cognitive abilities. ONH listeners can attend to the linguistic components of the message, while OHI listeners have reduced ability to gain access to the speech signal during glimpses. That is, sufficient cognitive resources for top-down linguistic processing are not available after glimpsing for OHI listeners [24]. This may indicate that OHI listeners engage in shallower depth of processing, whereas ONH listeners may have sufficient spare capacity after auditory processing to engage in more elaborate (i.e., linguistic) processing. An alternative interpretation is a difference in learned listening strategy, with OHI listeners more attuned than ONH listeners to focusing on the acoustic signal during speech recognition.

Overall, the results of this study are generally consistent with prior studies of individual differences in speech recognition among older adults showing auditory and cognitive contributions (e.g., [5,7,11,30]). The combination of four predictor variables indexing auditory and cognitive factors explained 73–89% of the total variance in degraded speech recognition with good generalization of the findings to other measures of speech recognition involving effects of attention, multitalker masking, and speech interruption.

Regarding auditory factors, consistent with [5] that also controlled effects of reduced speech audibility, the audiogram was a significant predictor of speech recognition; accounting here for 29–30% of the variance even among ONH listeners. Thus, when audibility is accounted for, elevated hearing thresholds may serve as a proxy for contributions of cochlear impairments to auditory processing, such as the loss or dysfunction of inner hair cells for individuals with high thresholds (e.g., above 60 dB HL [59]), reduced endocochlear potentials (e.g., [60,61]), or cochlear synaptopathy that may be associated with speech-in-noise deficits (e.g., [62]).

Regarding cognitive factors, and consistent with [5], working memory accounted for 16–18% of the variance to degraded speech processing for both ONH and OHI listeners. This is consistent with the Ease of Language Understanding model (ELU) [63], which suggests that working memory becomes more important when the speech signal is degraded. According to the ELU model, working memory is activated to match the degraded input with stored representations in memory. However, the importance of working memory did not appear to increase at poorer SRTs (see Fig 5), nor was it markedly different between ONH and OHI listeners. This suggests that, at least for these materials, OHI listeners do not recruit working memory to a greater extent than ONH listeners.

This study is limited in its use of prior datasets that were collected on the same participant sample. While this ensured predictor variables and test procedures were matched across studies, it is important to acknowledge that these datasets were not completely independent and that the size of the participant sample was limited. Increasing sample size and employing identical test procedures across different samples of participants would aid in the generalizability of these results to other listeners. However, the high consistency and substantial explanatory power of the results within this participant sample suggests that the obtained findings are robust and reliable.

## Conclusions

This study examined individual differences among ONH and OHI groups across three levels of speech recognition difficulty. Individual differences were assessed across three datasets that varied in spectral degradation and modulated noise masking. This multi-dataset approach provided for the investigation of different aspects of auditory and cognitive processing. Furthermore, results were generalized to other measures of speech recognition that modeled commonly assessed aspects of multi-talker masking, speaker segregation, and temporal interruption. These results demonstrated similarities in how ONH and OHI groups recruit auditory processing skills, indexed by the audiogram, and working memory when listening to degraded speech in noise. However, group differences suggest that ONH allocate more cognitive resources for processing the linguistic message, compared to OHI who are limited by reduced abilities to glimpse information from the degraded speech signal. The multi-dataset replication and generalization of analyses support the validity and robustness of these findings.

Overall, these results highlight the need to improve auditory access to the acoustic signal, even after spectral shaping, for OHI listeners who may have difficulty accessing speech information in degraded conditions. Enhanced auditory access may supply essential speech cues and reduce cognitive load, thereby enabling OHI listeners to engage in linguistic processing more effectively, as observed in ONH listeners.

## Supporting information

S1 Appendix Psychoacoustic modulation detection and interference tasks. (PDF)

S1 Supporting Information. Primary data and dominance analysis.
(XLSX)

## References

[1] International Organization for Standardization. ISO-7029, Acoustics-Statistical Distribution of Hearing Thresholds as a Function of Age. Basel, Switzerland: ISO. 2000.

[2] Committee on Hearing, Bioacoustics, and Biomechanics (CHABA). Speech understanding and aging. The Journal of the Acoustical Society of America. 1988;83(3):859–95. doi: 10.1121/1.395965 3281988

[3] Akeroyd MA. Are individual differences in speech reception related to individual differences in cognitive ability? A survey of twenty experimental studies with normal and hearing-impaired adults. Int J Audiol. 2008;47 Suppl 2:S53-71. doi: 10.1080/14992020802301142 19012113

[4] Houtgast T, Festen JM. On the auditory and cognitive functions that may explain an individual’s elevation of the speech reception threshold in noise. Int J Audiol. 2008;47(6):287–95. doi: 10.1080/14992020802127109 18569101

[5] Humes LE. Factors Underlying Individual Differences in Speech-Recognition Threshold (SRT) in Noise Among Older Adults. Front Aging Neurosci. 2021;13:702739. doi: 10.3389/fnagi.2021.702739 34290600 PMC8287901

[6] Humes LE, Dubno JR. Factors affecting speech understanding in older adults. The aging auditory system. San Diego: Plural Publishing. 2009. p. 211–57.

[7] Humes LE, Kidd GR, Lentz JJ. Auditory and cognitive factors underlying individual differences in aided speech-understanding among older adults. Front Syst Neurosci. 2013;7:55. doi: 10.3389/fnsys.2013.00055 24098273 PMC3787592

[8] Humes LE, Watson BU, Christensen LA, Cokely CG, Halling DC, Lee L. Factors associated with individual differences in clinical measures of speech recognition among the elderly. J Speech Hear Res. 1994;37(2):465–74. doi: 10.1044/jshr.3702.465 8028328

[9] Jerger J, Jerger S, Pirozzolo F. Correlational analysis of speech audiometric scores, hearing loss, age, and cognitive abilities in the elderly. Ear Hear. 1991;12(2):103–9. doi: 10.1097/00003446-199104000-00004 2065833

[10] Jerger J, Jerger S, Oliver T, Pirozzolo F. Speech understanding in the elderly. Ear Hear. 1989;10(2):79–89. doi: 10.1097/00003446-198904000-00001 2707505

[11] Rönnberg J, Lunner T, Ng EHN, Lidestam B, Zekveld AA, Sörqvist P, et al. Hearing impairment, cognition and speech understanding: exploratory factor analyses of a comprehensive test battery for a group of hearing aid users, the n200 study. Int J Audiol. 2016;55(11):623–42. doi: 10.1080/14992027.2016.1219775 27589015 PMC5044772

[12] van Rooij JC, Plomp R. Auditive and cognitive factors in speech perception by elderly listeners. II: Multivariate analyses. J Acoust Soc Am. 1990;88(6):2611–24. doi: 10.1121/1.399981 2283434

[13] van Rooij JC, Plomp R. Auditive and cognitive factors in speech perception by elderly listeners. III. Additional data and final discussion. J Acoust Soc Am. 1992;91(2):1028–33. doi: 10.1121/1.402628 1556304

[14] van Rooij JC, Plomp R, Orlebeke JF. Auditive and cognitive factors in speech perception by elderly listeners. I: Development of test battery. J Acoust Soc Am. 1989;86(4):1294–309. doi: 10.1121/1.398744 2808905

[15] Eckert MA, Teubner-Rhodes S, Vaden KI Jr, Ahlstrom JB, McClaskey CM, Dubno JR. Unique patterns of hearing loss and cognition in older adults’ neural responses to cues for speech recognition difficulty. Brain Struct Funct. 2022;227(1):203–18. doi: 10.1007/s00429-021-02398-2 34632538 PMC9044122

[16] Vaden KI Jr, Kuchinsky SE, Cute SL, Ahlstrom JB, Dubno JR, Eckert MA. The cingulo-opercular network provides word-recognition benefit. J Neurosci. 2013;33(48):18979–86. doi: 10.1523/JNEUROSCI.1417-13.2013 24285902 PMC3841458

[17] Adank P. The neural bases of difficult speech comprehension and speech production: Two Activation Likelihood Estimation (ALE) meta-analyses. Brain Lang. 2012;122(1):42–54. doi: 10.1016/j.bandl.2012.04.014 22633697

[18] Eckert MA, Walczak A, Ahlstrom J, Denslow S, Horwitz A, Dubno JR. Age-related effects on word recognition: reliance on cognitive control systems with structural declines in speech-responsive cortex. J Assoc Res Otolaryngol. 2008;9(2):252–9. doi: 10.1007/s10162-008-0113-3 18274825 PMC2504602

[19] Erb J, Obleser J. Upregulation of cognitive control networks in older adults’ speech comprehension. Front Syst Neurosci. 2013;7:116. doi: 10.3389/fnsys.2013.00116 24399939 PMC3871967

[20] Ohlenforst B, Zekveld AA, Jansma EP, Wang Y, Naylor G, Lorens A, et al. Effects of Hearing Impairment and Hearing Aid Amplification on Listening Effort: A Systematic Review. Ear Hear. 2017;38(3):267–81. doi: 10.1097/AUD.0000000000000396 28234670 PMC5405775

[21] Guang C, Lefkowitz E, Dillman-Hasso N, Brown VA, Strand JF. Recall of Speech is Impaired by Subsequent Masking Noise: A Replication of Experiment 2. Audit Percept Cogn. 2020;3(3):158–67. doi: 10.1080/25742442.2021.1896908 34240010 PMC8262135

[22] Rabbitt PMA. Channel-Capacity, Intelligibility and Immediate Memory. Quarterly Journal of Experimental Psychology. 1968;20(3):241–8. doi: 10.1080/14640746808400158 5683763

[23] Wingfield A, Tun PA, McCoy SL. Hearing Loss in Older Adulthood. Curr Dir Psychol Sci. 2005;14(3):144–8. doi: 10.1111/j.0963-7214.2005.00356.x

[24] Pichora-Fuller MK, Kramer SE, Eckert MA, Edwards B, Hornsby BWY, Humes LE, et al. Hearing Impairment and Cognitive Energy: The Framework for Understanding Effortful Listening (FUEL). Ear Hear. 2016;37 Suppl 1:5S-27S. doi: 10.1097/AUD.0000000000000312 27355771

[25] Powell DS, Oh ES, Lin FR, Deal JA. Hearing Impairment and Cognition in an Aging World. J Assoc Res Otolaryngol. 2021;22(4):387–403. doi: 10.1007/s10162-021-00799-y 34008037 PMC8329135

[26] Humes LE. The contributions of audibility and cognitive factors to the benefit provided by amplified speech to older adults. J Am Acad Audiol. 2007;18(7):590–603. doi: 10.3766/jaaa.18.7.6 18236646

[27] Dryden A, Allen HA, Henshaw H, Heinrich A. The Association Between Cognitive Performance and Speech-in-Noise Perception for Adult Listeners: A Systematic Literature Review and Meta-Analysis. Trends Hear. 2017;21:2331216517744675. doi: 10.1177/2331216517744675 29237334 PMC5734454

[28] Heinrich A. The role of cognition for speech-in-noise perception: Considering individual listening strategies related to aging and hearing loss. Int J Behav Dev. 2021;45(5):382–8. doi: 10.1177/01650254211000377

[29] Heinrich A, Henshaw H, Ferguson MA. The relationship of speech intelligibility with hearing sensitivity, cognition, and perceived hearing difficulties varies for different speech perception tests. Front Psychol. 2015;6:782. doi: 10.3389/fpsyg.2015.00782 26136699 PMC4468362

[30] Cherri D, Eddins DA, Ozmeral EJ. A Step Toward Precision Audiology: Individual Differences and Characteristic Profiles From Auditory Perceptual and Cognitive Abilities. Trends Hear. 2024;28:23312165241263485. doi: 10.1177/23312165241263485 39099537 PMC11301744

[31] Sanchez-Lopez R, Fereczkowski M, Neher T, Santurette S, Dau T. Robust Data-Driven Auditory Profiling Towards Precision Audiology. Trends Hear. 2020;24:2331216520973539. doi: 10.1177/2331216520973539 33272110 PMC7720332

[32] Wu Y-H, Stangl E, Zhang X, Perkins J, Eilers E. Psychometric Functions of Dual-Task Paradigms for Measuring Listening Effort. Ear Hear. 2016;37(6):660–70. doi: 10.1097/AUD.0000000000000335 27438866 PMC5079765

[33] Folstein MF, Folstein SE, McHugh PR. “Mini-mental state”. A practical method for grading the cognitive state of patients for the clinician. J Psychiatr Res. 1975;12(3):189–98. doi: 10.1016/0022-3956(75)90026-6 1202204

[34] Fogerty D, Ahlstrom JB, Dubno JR. Glimpsing keywords across sentences in noise: A microstructural analysis of acoustic, lexical, and listener factors. J Acoust Soc Am. 2021;150(3):1979. doi: 10.1121/10.0006238 34598610 PMC8448575

[35] Fogerty D, Ahlstrom JB, Dubno JR. Sentence recognition with modulation-filtered speech segments for younger and older adults: Effects of hearing impairment and cognition. J Acoust Soc Am. 2023;154(5):3328–343. doi: 10.1121/10.0023323 37983296 PMC10663055

[36] Nakagawa S. Krauklis wave propagation within a complex fracture system: Modeling via a two-dimensional time-harmonic boundary element method. J Acoust Soc Am. 2024;156(1):610–22. doi: 10.1121/10.0028006 39029096

[37] Institute of Electrical and Electronic Engineers. IEEE Recommended Practice for Speech Quality Measures. New York: IEEE. 1969.

[38] Bernstein JG, Grant KW. Auditory and auditory-visual intelligibility of speech in fluctuating maskers for normal-hearing and hearing-impaired listeners. J Acoust Soc Am. 2009;125(5):3358–72. doi: 10.1121/1.3097496 19425676

[39] Fogerty D, Ahlstrom JB, Bologna WJ, Dubno JR. Sentence intelligibility during segmental interruption and masking by speech-modulated noise: Effects of age and hearing loss. J Acoust Soc Am. 2015;137(6):3487–501. doi: 10.1121/1.4921603 26093436 PMC4474944

[40] Lachman ME, Agrigoroaei S, Tun PA, Weaver SL. Monitoring cognitive functioning: psychometric properties of the brief test of adult cognition by telephone. Assessment. 2014;21(4):404–17. doi: 10.1177/1073191113508807 24322011 PMC4050038

[41] Salthouse TA, Toth J, Daniels K, Parks C, Pak R, Wolbrette M, et al. Effects of aging on efficiency of task switching in a variant of the Trail Making Test. Neuropsychology. 2000;14(1):102–11. doi: 10.1037/0894-4105.14.1.102 10674802

[42] Robertson IH, Tegnér R, Tham K, Lo A, Nimmo-Smith I. Sustained attention training for unilateral neglect: theoretical and rehabilitation implications. J Clin Exp Neuropsychol. 1995;17(3):416–30. doi: 10.1080/01688639508405133 7650103

[43] Dunn LM, Dunn DM. Peabody Picture Vocabulary Test. 4th ed. Bloomington, MN: Pearson Assessments. 2007.

[44] Besser J, Koelewijn T, Zekveld AA, Kramer SE, Festen JM. How linguistic closure and verbal working memory relate to speech recognition in noise--a review. Trends Amplif. 2013;17(2):75–93. doi: 10.1177/1084713813495459 23945955 PMC4070613

[45] Fogerty D, Dubno JR, Shafiro V. Perception of interrupted speech and text: Listener and modality factors. JASA Express Lett. 2022;2(6):064402. doi: 10.1121/10.0011571 36154160 PMC9909681

[46] Garofolo J, Lamel L, Fisher W, Fiscus J, Pallett D, Dahlgren N. DARPA TIMIT Acoustic-Phonetic Continuous Speech Corpus CD-ROM. National Institute of Standards and Technology. 1993.

[47] Lorenzi C, Berthommier F, Apoux F, Bacri N. Effects of envelope expansion on speech recognition. Hear Res. 1999;136(1–2):131–8. doi: 10.1016/s0378-5955(99)00117-3 10511632

[48] Apoux F, Crouzet O, Lorenzi C. Temporal envelope expansion of speech in noise for normal-hearing and hearing-impaired listeners: effects on identification performance and response times. Hear Res. 2001;153(1–2):123–31. doi: 10.1016/s0378-5955(00)00265-3 11223303

[49] Saija JD, Akyürek EG, Andringa TC, Başkent D. Perceptual restoration of degraded speech is preserved with advancing age. J Assoc Res Otolaryngol. 2014;15(1):139–48. doi: 10.1007/s10162-013-0422-z 24198087 PMC3901857

[50] Shafiro V, Sheft S, Risley R. The intelligibility of interrupted and temporally altered speech: effects of context, age, and hearing loss. J Acoust Soc Am. 2016;139(1):455–65. doi: 10.1121/1.4939638 26827039 PMC4723407

[51] Freyman RL, Keen R. Constructing and disrupting listeners’ models of auditory space. J Acoust Soc Am. 2006;120(6):3957–65. doi: 10.1121/1.2354020 17225422

[52] Bolia RS, Nelson WT, Ericson MA, Simpson BD. A speech corpus for multitalker communications research. J Acoust Soc Am. 2000;107(2):1065–6. doi: 10.1121/1.428288 10687719

[53] Bilger RC, Nuetzel JM, Rabinowitz WM, Rzeczkowski C. Standardization of a test of speech perception in noise. J Speech Hear Res. 1984;27(1):32–48. doi: 10.1044/jshr.2701.32 6717005

[54] Kalikow DN, Stevens KN, Elliott LL. Development of a test of speech intelligibility in noise using sentence materials with controlled word predictability. J Acoust Soc Am. 1977;61(5):1337–51. doi: 10.1121/1.381436 881487

[55] Jensen J, Taal CH. An Algorithm for Predicting the Intelligibility of Speech Masked by Modulated Noise Maskers. IEEE/ACM Trans Audio Speech Lang Process. 2016;24(11):2009–22. doi: 10.1109/taslp.2016.2585878

[56] Azen R, Budescu DV. The dominance analysis approach for comparing predictors in multiple regression. Psychol Methods. 2003;8(2):129–48. doi: 10.1037/1082-989x.8.2.129 12924811

[57] Budescu DV, Azen R. Beyond Global Measures of Relative Importance: Some Insights from Dominance Analysis. Organizational Research Methods. 2004;7(3):341–50. doi: 10.1177/1094428104267049

[58] Miller RE, Gibbs BE II, Fogerty D. Glimpsing speech interrupted by speech-modulated noise. The Journal of the Acoustical Society of America. 2018;143(5):3058–67. doi: 10.1121/1.5038273 29857765 PMC5966307

[59] Moore BC. Dead regions in the cochlea: conceptual foundations, diagnosis, and clinical applications. Ear Hear. 2004;25(2):98–116. doi: 10.1097/01.AUD.0000120359.49711.D7 15064655

[60] Schmiedt RA, Lang H, Okamura H, Schulte BA. Effects of furosemide applied chronically to the round window: a model of metabolic presbyacusis. J Neurosci. 2002;22(21):9643–50. doi: 10.1523/JNEUROSCI.22-21-09643.2002 12417690 PMC6758027

[61] Eckert MA, Harris KC, Lang H, Lewis MA, Schmiedt RA, Schulte BA, et al. Translational and interdisciplinary insights into presbyacusis: A multidimensional disease. Hear Res. 2021;402:108109. doi: 10.1016/j.heares.2020.108109 33189490 PMC7927149

[62] Furman AC, Kujawa SG, Liberman MC. Noise-induced cochlear neuropathy is selective for fibers with low spontaneous rates. J Neurophysiol. 2013;110(3):577–86. doi: 10.1152/jn.00164.2013 23596328 PMC3742994

[63] Rönnberg J, Lunner T, Zekveld A, Sörqvist P, Danielsson H, Lyxell B, et al. The Ease of Language Understanding (ELU) model: theoretical, empirical, and clinical advances. Front Syst Neurosci. 2013;7:31. doi: 10.3389/fnsys.2013.00031 23874273 PMC3710434

