# Supplementary material for: Auditory and cognitive contributions to recognition of degraded speech in noise: Individual differences among older adults
Source: PLoS One. 2025 Sep 4;20(9):e0331487. doi: 10.1371/journal.pone.0331487 (PMC12410885; doi:10.1371/journal.pone.0331487)
Supplement: S1 Appendix — (PDF) [file pone.0331487.s001.pdf]

S1 Appendix.

Psychoacoustic modulation detection and interference tasks.

Methods. Stimuli consisted of an idealized standard speech-shaped noise [1] at a preprocessed level of 70 dB SPL, filtered into two one-third octave frequency bands centered at 500 Hz and 3150 Hz. Target noise bands were embedded in a broadband speech-shaped noise, notched at the target bands, and presented at a preprocessed level 20 dB below the calibrated target level. The noise stimuli were 500 ms in duration and shaped with 10-ms rise/fall raised cosine ramps. The noise bands were amplitude modulated by the Hilbert temporal envelope (low pass filtered at 16 Hz with a first-order Butterworth filter) of a single talker in the target frequency band. The modulation depth of the speech envelope, E(t), was varied using an exponential compression factor $E(t)^K$, followed by rescaling each band to the target presentation level. Stimuli were presented in a three-interval two-alternative forced choice task using an adaptive tracking procedure targeting 70.7% correct performance [2]. Thresholds for both modulation detection and interference were calculated by averaging the last six reversals after the minimum step size (0.05, starting step size = 0.4) was reached, using a Parameter Estimation by Sequential Testing (PEST) [3] adaptive method. Each condition (i.e., 500 Hz and 3150 Hz) included three blocks; the final threshold was defined by averaging the two closest values. Participants responded by pressing one of two buttons on a touch screen monitor that corresponded to the listening intervals. Training (with correct answer highlighted) and demo blocks (without highlighting) were provided before testing, and response feedback was given following all trials.

Data Reduction. The four measures of modulation detection and interference were also subjected to PCA to extract factors with eigenvalues greater than 1. A single component was

identified that explained 55% of the variance. Communalities were above .57 (KMO = .68), except for one communality below .4, for modulation interference at 500 Hz. This marginal fit was partially due to some independence in thresholds for the different target bands among the OHI listeners. Therefore, results were analyzed based on the four raw psychoacoustic speech modulation thresholds along with this summary component. As no difference in conclusions were made by including the raw scores, results for the single speech modulation PCA are reported here as a summary measure.

References

1. American National Standards Institute. Methods for calculation of the Speech Intelligibility Index (ANSI S3.5-1997 [R2007]). New York: American National Standards Institute; 2007.
2. Levitt H. Transformed up-down methods in psychoacoustics. J Acoust Soc Am. 1971;49(2B):467-477. doi:10.1121/1.1912375.
3. Taylor M, Creelman CD. PEST: Efficient estimates on probability functions. J Acoust Soc Am. 1967;41(4):782-787. doi:10.1121/1.1910407.
